# Transcriptional Regulation of *Fucosyltransferase 1* Gene Expression in Colon Cancer Cells

**DOI:** 10.1155/2013/105464

**Published:** 2013-03-03

**Authors:** Fumiko Taniuchi, Koji Higai, Tomomi Tanaka, Yutaro Azuma, Kojiro Matsumoto

**Affiliations:** Department of Medical Biochemistry, Faculty of Pharmaceutical Sciences, Toho University, Miyama 2-2-1, Funabashi, Chiba 274-8510, Japan

## Abstract

The **α**1,2-fucosyltransferase I (FUT1) enzyme is important for the biosynthesis of H antigens, Lewis B, and Lewis Y. In this study, we clarified the transcriptional regulation of FUT1 in the DLD-1 colon cancer cell line, which has high expression of Lewis B and Lewis Y antigens, expresses the *FUT1* gene, and shows **α**1,2-fucosyltransferase (FUT) activity. 5′-rapid amplification of cDNA ends revealed a FUT1 transcriptional start site −10 nucleotides upstream of the site registered at NM_000148 in the DataBase of Human Transcription Start Sites (DBTSS). Using the dual luciferase assay, *FUT1* gene expression was shown to be regulated at the region −91 to −81 nt to the transcriptional start site, which contains the Elk-1 binding site. Site-directed mutagenesis of this region revealed the Elk-1 binding site to be essential for FUT1 transcription. Furthermore, transfection of the dominant negative Elk-1 gene, and the chromatin immunoprecipitation (CHIp) assay, supported Elk-1-dependent transcriptional regulation of *FUT1* gene expression in DLD-1 cells. These results suggest that a defined region in the 5′-flanking region of FUT1 is critical for FUT1 transcription and that constitutive gene expression of *FUT1* is regulated by Elk-1 in DLD-1 cells.

## 1. Introduction

Incomplete synthesis and neosynthesis are two major factors affecting cancer-associated alterations of cell surface carbohydrate determinants. Expression of sialyl Lewis A/X, NeuAc*α*2,3Gal*β*1,3/4(Fuc*α*1,4/3)GlcNAc-R, is accelerated in the advanced stages of cancer by hypoxia-induced transcription of several glycogenes [1–3]. In normal epithelial cells, disialyl Lewis A, NeuAc*α*2,3Gal*β*1,3(NeuAc*α*2,6)GlcNAc-R, and sialyl 6-sulfo Lewis X, NeuAc*α*2,3Gal*β*1,4 (SO_3_-6) GlcNAc-R, are preferentially expressed; they are synthesized by GlcNAc: *α*2,6-sialyltransferase (ST6GlcNAc) VI [4] and GlcNAc: *β*6-sulfotransferase (6-SulT) [5], respectively.

Blood group A and B antigens expressed on leukocytes and epithelial cells are synthesized by the transfer of GalNAc and Gal to precursor H antigen, Fuc*α*1,2Gal*β*1-R, catalyzed by A-transferase and B-transferase and producing A antigen, GalNAc*α*1,3(Fuc*α*1,2)Gal*β*1-R, and B antigen, Gal*α*1,3(Fuc*α*1,2)Gal*β*1-R, respectively. This process is significant during the development, differentiation, and maturation of normal cells [6]. ABH antigens are deleted or reduced in various cancers including myeloid malignancies [7], leukemia [8], oral cancer [9], and bladder cancer [10]. The A-transferase gene promoter region contains CpG-rich sites whose methylation status correlates well with gene expression; treatment with 5-aza-dC results in the appearance of A-transferase gene and A-antigen expression [11]. The Sd^a^ blood group carbohydrate structure, GalNAc*β*1,4(NeuAc*α*2,3)Gal*β*1,4GlcNAc-R, and *β*1,4-GalNAc transferase (*β*1,4-GalNAcT) II, which is responsible for Sd^a^ synthesis gene expression, are abundantly expressed in the normal gastrointestinal mucosa, while their expression levels are markedly decreased in gastric and colonic cancers. Treatment of cancer cells with 5-aza-dC induces expression of Sd^a^ and *β*1,4-GalNAcT II and reduces their metastatic potential [12–14].

Loss of H antigen in myeloid malignancies and leukemia [7, 8] and loss of *α*1,2-fucosyltransferase (FUT) activities in gastric cancer [15] are common, although the mechanism remains to be elucidated. FUT1 (H enzyme) and FUT2 (Se enzyme) genes are cloned and responsible for synthesis of *α*1,2-fucosylation on both core structures of type 1/2, Gal*β*1,3/4GlcNAc-R, producing H antigen and Lewis B/Y, Fuc*α*1,2Gal*β*1,3/4(Fuc*α*1,4/3)GlcNAc-R [16, 17]. H antigen, Lewis B, and Lewis Y are expressed in fetal distal colorectal mucosa, but not in adult tissues; they are reexpressed in colorectal carcinoma [18]. The stage- and tissue-specific expression of the *FUT1* gene is regulated by three transcriptional start sites (E1, E2, and E7) and alternative use of multiple promoters [19, 20]. It has been suggested that transcription starts from both E1 and E7 in an undifferentiated colorectal cancer cell line (SW-620); it was dramatically decreased at E1 but not E7 after differentiation of the cells by treatment with butyrate [20].

Here, we show transcriptional regulation of FUT1 in DLD-1 cells using 5′-rapid amplification of cDNA ends (5′-RACE), a dual luciferase assay for sequential deletion and site-directed mutagenesis, transient overexpression of dominant negative form of Elk-1, and chromatin immunoprecipitation (CHip) assay. Our results indicate that 5′-flanking regions at positions −91 to −81 nt relative to the *FUT1* gene transcription start site are critical for FUT1 transcription and mRNA expression in DLD-1 cells.

## 2. Materials and Methods

### 2.1. Cells and Cell Culture

 Human colorectal DLD-1 and SW48 cells were purchased from the Japanese Collection of Research Bioresources Cell Bank (Tokyo, Japan) and cultured in Dulbecco's modified Eagle's medium (DMEM) (Nissui Pharmaceutical Co., Tokyo, Japan) supplemented with 10% heat-inactivated fetal bovine serum (JRC Biosciences, Lenexa, KS, USA) under a humidified atmosphere containing 5% CO_2_ at 37°C.

### 2.2. Flow Cytometry Analysis

The primary mouse antibodies used in this study were obtained from Seikagaku Co. (Tokyo, Japan), including anti-Lewis B (IgG1, 2-25LE) and anti-Lewis Y (IgG3, 12-4LE). For determination of the carbohydrate antigens on the cell surface, cells (5 × 10^6^ cells/mL) were treated with primary antibodies (100 *μ*L of 1 *μ*g/mL of each) in phosphate-buffered saline (PBS) containing 1% bovine serum albumin (BSA) and 0.1% NaN_3_ for 1 h at 37°C and washed three times with 1% BSA/PBS. The cells were further incubated with fluorescein-isothiocyanate- (FITC-) conjugated anti-mouse IgG + IgM goat antibody (100 *μ*L, 1 *μ*g/mL) (SouthernBiotech, Birmingham, AL, USA) in 1% BSA/PBS for 1 h at 37°C. After washing three times with 1% BSA/PBS, the cells were subjected to flow cytometry using CytoAce300 (Jasco, Tokyo, Japan).

### 2.3. Measurement of *α*1,2-FUT Activity

*α*1,2-FUT activity was determined as described previously [21, 22]. Briefly, the cells were sonicated on ice three times for 10 s in Tris-HCl (10 mM, pH 7.0) containing 1% Triton X-100. After centrifugation, cell lysate (100 *μ*g protein, 20 *μ*L) was incubated with 0.5 mM Gal*β*1,4GlcNAc,which was aminated and coupled with 2-aminopyridyl-*β*-alanine (Wako, Osaka, Japan) in the presence of guanosine 5′-diphospho-*α*-L-fucose (GDP-Fuc; 0.5 mM; Sigma-Aldrich, St Louis, MO, USA), MglC_2_ (10 mM) in HEPES buffer (100 mM, pH 7.5) for 8 h at 37°C. After the reaction mixture was diluted with distilled water, boiled for 3 min, and centrifuged at 16,000 g for 3 min, an aliquot was injected onto Wakopak Handy ODS (4.5 mm id × 150 mm, Wako) at 55°C and the product was separated with a 1.0 mL/min flow rate of 2% acetonitrile in ammonium acetate (10 mM). The eluent was monitored by a Model FS-8020 fluorescence detector (Tosoh Co., Tokyo, Japan) at Ex 330 nm and Em 450 nm. The peak areas were calculated using a Model LC-8020 Multi Station (Tosoh).

### 2.4. RT-PCR

Total RNA was extracted from DLD-1 and SW48 cells using TRIzol reagent (Life Technologies Co., Carlsbad, CA, USA). The first-strand cDNA (20 *μ*L) was synthesized from total RNA (5 *μ*g) using ReverTra Ace reverse transcriptase (Toyobo, Tokyo, Japan) and oligo (dT)_20_ primer, followed by DNase I treatment, according to the manufacturer's instructions. cDNA (0.5 *μ*L) was amplified in a PC-812 thermal cycler (Astec Co., Fukuoka, Japan) using Go Taq (Promega, Madison, WI, USA) and specific forward and reverse primer sets, according to the manufacturer's instructions. The PCR conditions for FUT1 were 95°C for 2 min followed by 30 cycles of 98°C for 20 s, 60°C for 5 s, and 72°C for 30 s; for GAPDH we used 20 cycles. The specific forward and reverse primer sets used were as follows: 5′-GCAGCTTCACGACTGGATGTCGGAG-3′ and 5′-TACACCACTCCATGCCGTTGCTGGTGACCA-3′ for FUT1; 5′-CCACCCATGGCAAATTCCATGGCA-3′ and 5′-TCTAGACGGCAGGTCAGGTCCACC-3′ for GAPDH, respectively. Primer sequences were designed using Primer Express software version 2.0.0 (Applied Biosystems, Foster City, CA, USA).

### 2.5. Determination of the FUT1 Transcription Start Site in DLD-1 Cells Using 5′-Rapid Amplification of cDNA Ends (5′-RACE)

The 5′ end of FUT1 cDNA was amplified with 5′-Full RACE Core Set (Takara Bio Inc., Otsu, Japan) according to the manufacturer's instructions. First-strand cDNA was synthesized from 1 *μ*g of total RNA using the 5′-phosphorylated FUT-1-specific primer 5′-GATCGGGGATGCAGGGG-3′. Template mRNA was digested with RNase H at 37°C for 30 min and the cDNA precipitated by the addition of ethanol. The single-strand DNA precipitate was dissolved into the ligation buffer and incubated with T4 ligase at 16°C for 16 h. The concatemer DNA was used as a template for the first PCR amplification, using forward primer 5′-CCTTTGTCTCTGGAGCCG-3′ and reverse primer 5′-GGCTAACGTAGGGTCCAGCT-3′. PCR conditions were 94°C for 3 min followed by 25 cycles of 95°C for 30 s, 60°C for 30 s, and 72°C for 60 s. The resulting PCR products were diluted 100-fold with distilled water and amplified under the conditions described above, using the second forward primer 5′-CTCCAGCCTTGGAATGGTT-3′ and reverse primer 5′-AACCTGTCTTCCCTCTGGGT-3′. PCR products were ligated into pGL4 vector (Life Technologies) and sequenced using a 3730xl DNA analyzer (Applied Biosystems).

### 2.6. Deletion Constructs of Plasmids and Luciferase Assay

The 5′-flanking region −700 to +1 nt of the FUT1 transcriptional start site was amplified from DLD-1-derived genomic DNA by PCR using the forward and reverse primers 5′-AGGTGAGTAGACTCAGGTGGCCTG-3′ and 5′-CCCAGGTTCTTTCAGGAGCAC-3′, respectively. The PCR products were 5′-phosphorylated using T4 Polynucleotide Kinase (Toyobo) and ligated into pGL4 vector which was digested with *EcoRV* (Toyobo) and alkaline phosphatase from *E. coli* (Toyobo). The sequence was ascertained using a 3730xl DNA analyzer.

 Plasmids for pGL4.11/−700_+1, pGL4.11/−419_+1, pGL4.11/−190_+1, pGL4.11/−121_+1, pGL4.11/−100_+1, pGL4.11/−91_+1, and pGL4.11/−81_+1 were constructed by PCR, using pGL4/−700_+1 plasmid as the PCR template, and ligated into pGL4.11 vector. PCR conditions were 94°C for 2 min, followed by 30 cycles of 95°C for 0.5 min, 60°C for 5 min, and 72°C for 1 min. The specific forward primers used were 5′-AAGGATCTGGGGTCTCAAGG-3′ (pGL4.11/−419_+1), 5′-AGTATCCTGCCTTGGAGCC-3′ (pGL4.11/−190_+1), 5′-ATGCTCAGACCCTGGACATC-3′ (pGL4.11/−121_+1), 5′-TGGACATCCCAGCCTCCT-3′ (pGL4.11/−100_+1), 5′-AGCCTCCTCCTCCCTGATG-3′ (pGL4.11/−91_+1), and 5′-GCAATCCTGGTGTTTCTTTCA-3′ (pGL4.11/−81_+1), respectively. The specific reverse primer used was 5′-ATCCTCGAGGCTAGCG-3′. 

For the FUT1 promoter assay, 0.2 *μ*g of pGL4.11 constructs and 0.02 *μ*g of pRL-TK-Luc vector (Promega Co., Madison, WI, USA) plasmids were transiently cotransfected into DLD-1 cells (1–3 × 10^5^ cells per well) using Lipofectamine 2000 reagent (Life Technologies). The pRL-TK-Luc vector contains the TK promoter 5′-upstream of the *Renilla* luciferase gene. After 24 h, the cells were harvested and lysed. Firefly and *Renilla* luciferase activities were determined using the Dual Luciferase Assay System and 20/20n luminometer (Promega).

### 2.7. Site-Directed Mutagenesis of FUT1 Promoter Regions

 Site-directed mutagenesis of the 5′-flanking region of the *FUT1* gene was carried out using the KOD-Plus-Mutagenesis Kit (Toyobo), which is based on inverse-PCR. The pGL4.11/−190_+1 plasmid was used as PCR template. Primers used for site-directed mutagenesis were 5′-**TCGAT**TCCTCCCTGATGCAATCCTGGT-3′ and 5′-CTGGGATGTCCAGGGTCTGA-3′ for −190_+1_mut. PCR was performed according to the manufacturer's instructions. PCR products were digested with *DpnI* and self-ligated with DNA ligase. The recombinant plasmids were transformed into *E. coli DH5a* competent cells (Toyobo) and positive clones were confirmed by DNA sequencing using a 3730xl DNA analyzer. Dual luciferase activities were determined using the Dual Luciferase Assay System and 20/20n luminometer (Promega).

### 2.8. Transfection of the Dominant Negative Elk-1 Gene

A dominant negative Elk-1 (DN-Elk-1) vector designed for double-mutated Elk-1 S383A and S389A was prepared from Elk-1 vector (Toyobo) by inverse PCR using the KOD-mutagenesis kit according to the manufacturer's instructions. The DN-Elk-1 expression vector was amplified by PCR using the forward and reverse primers 5′-GTGCCCCGGCCAAGCTCT-3′ and 5′-GGGGCGCAATGGGAGCCAGGGTGCTCCAGAA-3′, respectively. Then, 5, 10, and 20 *μ*g of DN-Elk-1 vector was transfected into DLD-1 cells using Lipofectamine 2000 reagent. After 24 h transfection, total RNA was extracted and FUT1 and GAPDH mRNA levels were determined by RT-PCR.

The cDNA (5 *μ*L) was then used for quantitative RT-PCR using TaqMan Gene Expression Assay (Life Technologies), Probe qPCR (Toyobo), and the ABI Prism 7500Fast Detection system (Applied Biosystems) in a 96-well plate according to the manufacturer's instructions. The specific forward and reverse oligonucleotide primers used were assay No. Hs00355741_m1 for FUT1 and Hs03929097_g1 for GAPDH.

PCR conditions were 95°C for 10 min, followed by 40 cycles of 95°C for 30 s, 60°C for 60 s, and 72°C for 60 s. The amount of *FUT VI* transcript was determined for each sample and normalized to *GAPDH* levels.

### 2.9. Chromatin Immunoprecipitation Assay

The chromatin immunoprecipitation assay for the 5′-flanking region of the *FUT1* gene was performed using HaloCHIP System (Promega) according to the manufacturer's instructions. Human Elk-1 full-length cDNA that had been PCR-amplified from Elk-1 vector (Toyobo) was cloned into the pHTC vector (Promega) using the In-Fusion HD Cloning Kit (TaKaRa) with the forward and reverse primer sets 5′-ATTCCTACCGCGGATATGGACCCATCTGTGACG-3′ and 5′-GGCCCAAATCTAGATGGCTTCTGGGGCCCTGGG-3′, respectively. The recombinant plasmids were transformed into *E. coli* DH5*α* competent cells and positive clones were confirmed using a 3730xl DNA analyzer. 

DLD-1 cells were seeded at a density of 4–8 × 10^5^ cells per well of a 6-well plate and then transfected with 30 *μ*g of Halo-tagged Elk-1 expression plasmid using the Neon Transfection System (Life Technologies) with pulse voltage 1150 V, pulse width 30, pulse no 2. After 24 h, the cells were crosslinked with formaldehyde-containing PBS, then lysed in cold Mammalian Lysis Buffer and sonicated on ice six times for 10 s. After centrifuging the sonicated samples at 14,000 g for 5 min, the supernatant was precipitated with HaloLink Resin, washed, and released into supernatant with Reversal Buffer. After the eluted DNA was purified with Wizard SV Gel and PCR Clean up system (Promega), target regions were amplified by PCR using Go Taq (Promega) with specific primers according to the manufacturer's instructions. The specific forward and reverse oligonucleotide primers used were 5′-AACCTCAACCTCATCTGTCC-3′ and 5′-GGTTCTCTGGTGAAAGAAA-3′ for from −140 to −41 nt. of the 5′-flanking region of the *FUT1* gene, and 5′-TGATGTAACCTGGGGTCCTT-3′ and 5′-TGAGACTCAGGAATGTGGGC-3′ for −534 to −424 nt. The PCR conditions were 95°C for 2 min followed by 30 cycles of 98°C for 20 s, 50°C for 10 s and 72°C for 30 s. PCR products were separated by 3% agarose gel electrophoresis and amplified DNAs were detected with ethidium bromide.

## 3. Results and Discussion

### 3.1. Expression of Lewis B and Lewis Y Antigens, *α*1,2-FUT Activity, and FUT1 mRNA in DLD-1 and SW48 Cells

**Figure 1:**
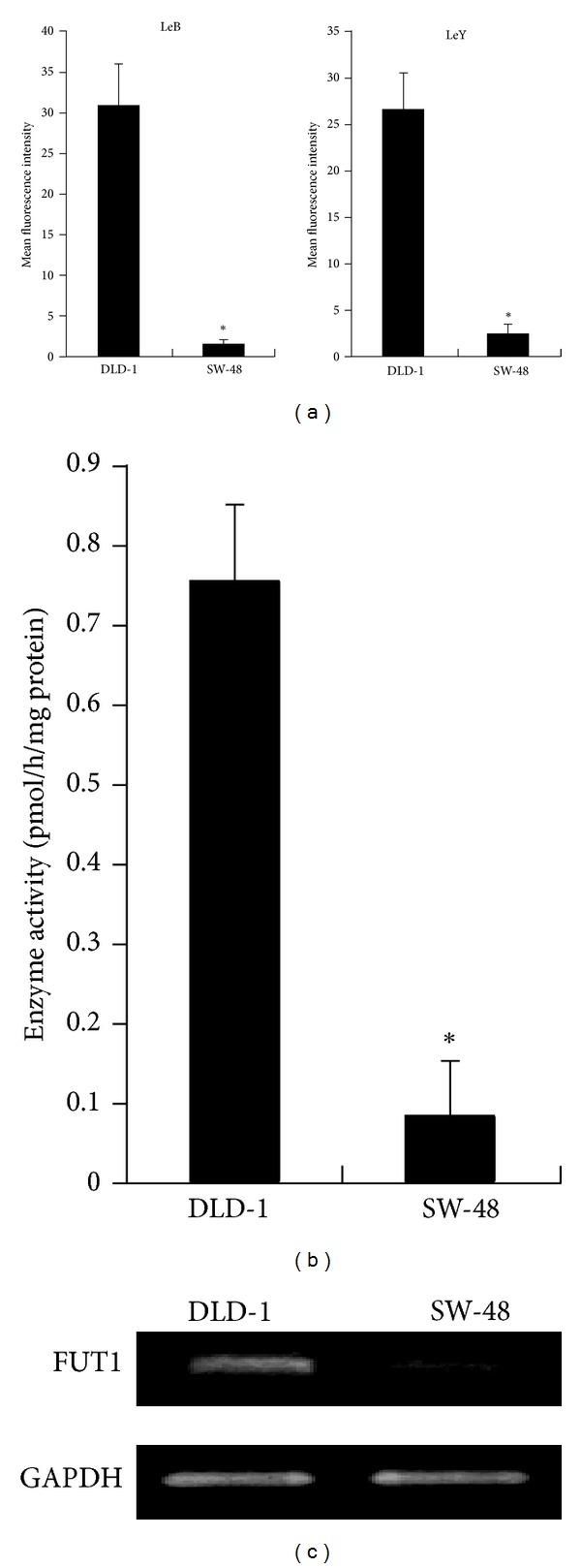
Expression of Lewis B/Y antigens, *α*1,2-FUT activities, and FUT1 mRNA in SW48 and DLD-1 cells. (a) Lewis B and Lewis Y antigens on the surface of SW48 and DLD-1 cells were determined using flow cytometry. (b) *α*1,2-FUT activities were determined by HPLC using fluorescent substrate Gal*β*1,4GlcNAc-CM. (c) FUT1 mRNA expression levels were determined by RT-PCR. The results were obtained in three independent experiments and data represent mean ± SD. Asterisks indicate significant differences (*P* < 0.05).

To clarify the regulation mechanism of the carbohydrate phenotypes, we determined Lewis B and Lewis Y carbohydrate antigens on two colorectal carcinoma cell lines using flow cytometry. Both Lewis B and Lewis Y antigens were abundantly expressed on DLD-1 cells, while expression levels on SW48 cells were low (Figure 1(a)). The *α*1,2-FUT activities responsible for synthesis of these carbohydrate antigens, determined using fluorescence substrate Gal*β*1,4GlcNAc-CM, were low in SW48 cells compared with DLD-1 cells (Figure 1(b)). RT-PCR revealed that FUT1 mRNA expression levels were also increased in DLD-1 cells compared with SW48 cells (Figure 1(c)). These results indicate that FUT1 mRNA expression levels are variable in colorectal cancer cell lines and correlate well with the expression levels of Lewis B and Lewis Y antigens.

### 3.2. Promoter Activities of Deletion Constructs of the FUT1 5′-Flanking Region

**Figure 2:**
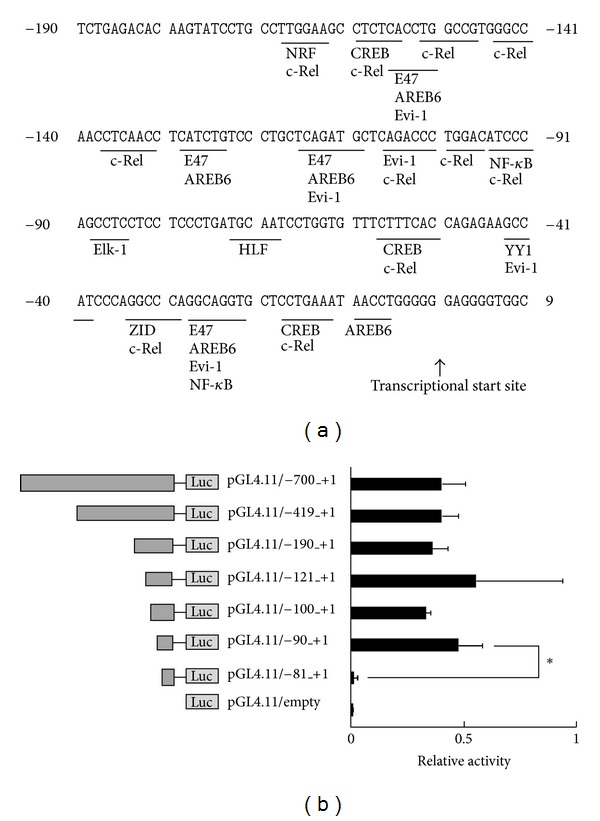
Nucleotide sequence of the 5′-flanking region of the *FUT1* gene and promoter activities of the FUT1 reporter gene in DLD-1 cells. (a) Consensus transcription-factor-binding motifs are detectable in the 5′-flanking region of FUT1. Nucleotides are numbered relative to the transcription start site obtained from 5′-RACE analysis (i.e., the observed transcription start site was set to +1). (b) FUT1 promoter deletion constructs and their luciferase activities in DLD-1 cells. Luciferase reported plasmids were constructed from DLD-1 genomic DNA by PCR and ligation. In each case, each plasmid construct and pRL-CMV were cotransfected into DLD-1 cells and luciferase activity was determined in a dual-luciferase assay 24 h aftertransfection. Firefly luciferase activities were normalized to *Renilla* luciferase activity to correct for differences in transfection efficiency. The results obtained in three independent experiments are expressed as mean ± SD. Significant differences (*P* < 0.05) are indicated by asterisks.

To clarify the highly constitutive expression of the *FUT1* gene in DLD-1 cells, we determined its transcription start site using 5′-RACE, which revealed one major transcript of about 400 bp (data not shown), indicating that the *FUT1* gene was transcribed at the E1 promoter [19, 20]. Based on sequence analysis, this gene product was transcribed −10 nt from the site registered at NM_000148 in the DataBase of Human Transcription Start Sites (DBTSS). The transcription start site and 5′-untranscribed region of FUT1 genomic DNA are shown in Figure 2(a). A homology search using the Match program (http://www.gene-regulation.com/index.html) revealed that the 5′-untranscribed region (−190 to +1) of the FUT1 gene contained several putative binding sites for transcription factors such as Elk-1, c-Rel, NF-*κ*B, AREB6, and CREB.

To identify transcriptional regulation of the *FUT1* gene in DLD-1 cells, we prepared FUT1 promoter deletion constructs using pGL4/−700_+1 as PCR template and ligated them into pGL4.11. After cotransfection of these deletion constructs with pRL-TK-Luc vector into DLD-1 cells, dual luciferase activities were determined (Figure 2(b)). Firefly luciferase activities were markedly decreased on deletion of the −91 to −81 nt region. This region contained a consensus Elk-1 binding site, indicating that transcription of the *FUT1* gene in DLD-1 cells was constitutively regulated by Elk-1.

### 3.3. Site-Directed Mutagenesis of FUT VI

**Figure 3:**
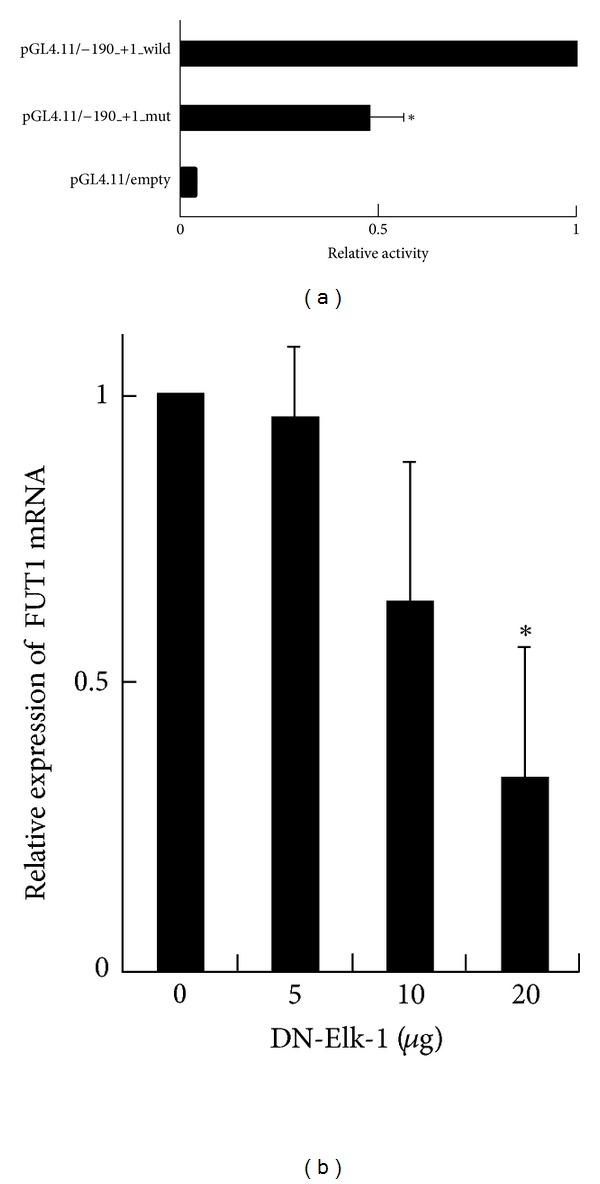
Site-directed mutagenesis on *FUT1* promoter and effect of DN-Elk-1 overexpression on expression of FUT1 mRNA in DLD-1 cells. (a) Mutagenesis in the region −90 to −81 of the *FUT1 *promoter. 10 *μ*g of the wild (pGL4.11/−190_+1_wild) and mutant (pGL4.11/−190_+1_mut) constructs and 0.5 *μ*g of pRL-CMV were co-transfected into DLD-1 cells and luciferase activity was determined 24 h aftertransfection. In each case, firefly luciferase activity was normalized to *Renilla* luciferase activity to correct for differences in transfection efficiency. The relative activities of luciferase are normalized to the activities of pGL4.11/−190_+1_wild as 100. The results obtained in three independent experiments are expressed as mean ± SD. Significant differences (*P* < 0.05) compared with the equivalent wild-type reporter construct are indicated by asterisks. (b) FUT1 mRNA expression in DN-Elk-1-transfected cells. After transfection with 5, 10, and 20 *μ*g DN-Elk-1 expression vector, FUT1 mRNA expression levels in DLD-1 cells were determined by RT-PCR. The results were obtained in three independent experiments and data represent mean ± SD. Significant differences (*P* < 0.05) compared with non-transfected cells are indicated by asterisks.

**Figure 4:**
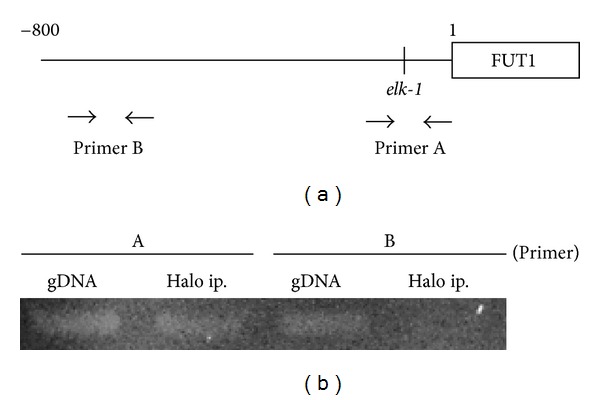
Chromatin immunoprecipitation analysis of FUT1 promoter in DLD-1 cells. Chromatin immunoprecipitation (CHIp) analysis for the Elk-1 binding site region within FUT1 promoter in DLD-1 cells. (a) Primers designed for the 5′-flanking region of FUT1 (Primer A: −140 to −41, Primer B: −534 to −424). (b) DLD-1 cells transfected with pHTC/Elk-1 vector were precipitated and amplified by PCR with specific primers (see (a)) as described in Section 2. gDNA: positive control; PCR amplification for fragmented and nonprecipitated genomic DNA from DLD-1 cells 24 h aftertransfection of Halo-tagged Elk-1 gene. Halo ip: PCR amplification for fragmented and precipitated genomic DNA with Halo resin from the DLD-1 cells 24 h aftertransfection of Halo-tagged Elk-1 gene.

To determine whether Elk-1 can upregulate FUT1 gene transcription, we prepared mutated constructs (AG**T**C**GAT**TCC) of the −90 to −81 nt region. The mutant constructs were then transfected into DLD-1 cells and the luciferase activity of each was determined (Figure 3(a)). In terms of the −186 to −156 nt region (Figure 4(a)), promoter activities of constructs carrying a four-base substitution (pGL4/−190_+1_mut) were significantly lower than that observed for the unmodified reporter construct (pGL4/−190_+1_wild). Although this suppression was incomplete, the promoter regions in the −90 to −81 region were important at least for FUT1 transcription.

### 3.4. Regulation of FUT1 Gene Expression by Elk-1

To confirm the transcriptional regulation of *FUT1* gene expression by Elk-1, we used RT-PCR to analyze the effect of overexpression of dominant negative (DN)-Elk-1 on FUT1 mRNA level. Phosphorylation at S383 and S389 of Elk-1 is essential for its transcriptional activity [23]. We transfected the DN-Elk-1, mutated S383A and S389A into DLD-1 cells and determined FUT1 mRNA expression using RT-PCR. FUT1 mRNA expression in DLD-1 cells was suppressed in a dose-dependent manner by 48 h transfection of the DN-Elk-1 gene (Figure 3(b)). These results indicate that constitutive FUT1 mRNA expression in DLD-1 cells is transcriptionally regulated by Elk-1.

### 3.5. Binding of the Elk-1 to the Promoter of FUT1

 To clarify whether Elk-1 regulates FUT1 mRNA expression, we determined its binding to the promoter of FUT1 using the CHip assay (Figure 4). We selected two primers, primer A for the promoter region of the 5′-flanking region of the *FUT1* gene and primer B for −534 to −424 nt (Figure 4(a)). Cells transfected with Halo-tagged Elk-1 gene were collected and the proteins cross-linked to the DNA, sonicated, and precipitated with Halo-resin. After the isolated DNA was amplified with PCR, the DNA bound to Elk-1 was visualized. Although the promoter region (−140 to −41) containing the Elk-1 binding site was precipitated with Elk-1, the FUT1 5′-flanking region (−534 to −424), with no Elk-1 binding site, was not (Figure 4(b)).

 In this report, we have confirmed that the transcriptional start site of FUT1 is located −10 nucleotides upstream of the site registered at NM_000148, and that constitutive expression of the *FUT1* gene is transcriptionally regulated by Elk-1, as confirmed by transfection of the DN-Elk-1 gene, site-directed mutagenesis, and the CHIp assay in DLD-1 cells. Expression of the ETS-like transcription factor Elk-1 gene is regulated by TATA box and the Erg-1 binding site, which functions specifically in monocytes [24]. Many glycosyltransferase genes have been reported to be regulated by Est family transcription factors, including GlcNAcT V [25–30], FUT4 [31], GalT I [32], GalT V [33], ST3Gal IV [34], and ST6GalNAc I [35].

Increased expression of *α*1,2-fucosylated glycans on the surface of rat colon carcinoma cells on transfection with the *FUT1* gene is associated with tumorigenicity and an increased resistance to apoptosis [36] and lymphokine activated killer cytotoxicity, but not to natural killer cell lysis [37]. Suppression of *FUT1* and *FUT4* gene expression by the short interfering RNA technique reduces Lewis Y expression and inhibits cell proliferation by decreasing the epidermal growth factor receptor signaling pathway and cancer growth [38]. Transfection of the *FUT1* gene in tumor cells selectively inhibits sialyl Lewis X and binding to E-selectin without affecting synthesis of sialyl Lewis A and binding to P-selectin [39, 40].

In tumor cells, incomplete synthesis of glycans results in reduced A and B blood antigens [7–11], disialyl Lewis A and sialyl 6-sulfo Lewis X antigens [41, 42] and Sd^a^ antigen [12–14] accompanied by the appearance or increase of H antigen, sialyl Lewis A and sialyl Lewis X antigens, and T, Tn and sialyl Tn antigens [3, 43]. Hypermethylation in the promoter region is involved in downregulation of A/B transferase [9, 11], ST6GlcNAc VI [41] and 6-SulT [42]. We also demonstrated in SW48 cells that many glycosyltransferase genes were downregulated, and that 5-aza-dC treatment enhanced FUT2, FUT4, FUT6, C2GnT, ST3Gal I, ST3Gal II, and ST3Gal IV mRNA expression (data not shown). However, epigenetic regulation of carbohydrate antigen synthesis in cancer cells would be more complicated.

In this report, we have suggested that the constitutive gene expression of *FUT1* is regulated at 5′-flanking regions at positions −91 to −81 nt of *FUT1* and that *FUT1* gene expression is upregulated by Elk-1 in DLD-1 cells. Further studies are needed to clarify the mechanism of expression of cancer-associated carbohydrate antigens with respect to direct regulation of glycosyltransferase genes and indirect regulation through expression of transcriptional factors.
